# Supplementary material for: Simplifying SARS-CoV-2 wastewater-based surveillance using an automated FDA EUA assay
Source: Microbiol Spectr. 2025 Feb 25;13(4):e02490-24. doi: 10.1128/spectrum.02490-24 (PMC11960137; doi:10.1128/spectrum.02490-24)
Supplement: Supplemental material — Supplemental methods, tables, and figures. [file spectrum.02490-24-s0001.docx]

**Supplemental Material**

**Supplemental Methods:**

**Quantitative reporting using the *m*2000 RealTi*m*e System (Abbott) SARS-CoV-2 EUA PCR assay.** We tested SeraCare calibration material provided at concentrations of 3.0, 4.0 and 5.0 log copies/mL to create an initial calibration curve to convert cycle threshold (Cq) values to log copies/mL (Supplemental Methods Table 1 and Figure 1). A six-member dilution series (serial ten-fold dilutions with dilution factors spanning 10 to 1,000,000) of pooled positive patient samples from clinical testing was subsequently tested with the initial SeraCare calibration applied. Dilutions with measured concentrations within the approximate 3.0-5.0 log copies/mL measuring range were quantified and after applying dilution factors, an established value was determined for the undiluted pooled positive sample and the six dilutions. An extended calibration curve spanning 2.9 to 7.9 log copies/mL was then created based on the assigned values of these dilutions and their Cq values (Supplemental Methods Table 2 and Figure 2). This extended calibration curve was then applied to all subsequent positive samples from the m2000 SARS-CoV-2 assay. The utility of this calibration curve in low concentration wastewater samples was confirmed in wastewater specimens by spiking SeraCare reference material into negative clarified wastewater samples to concentrations of 100, 50 and 25 copies/mL (Supplemental Methods Table 3).

**Supplemental Methods Table 1**. Cycle threshold values from quantitative reference material

| Seracare panel member | log cp/ml | m2000 Cq |
| --- | --- | --- |
| SeraCare P1 | 5.00 | 16.01 |
| SeraCare P2 | 4.00 | 19.30 |
| SeraCare P3 | 3.00 | 22.74 |


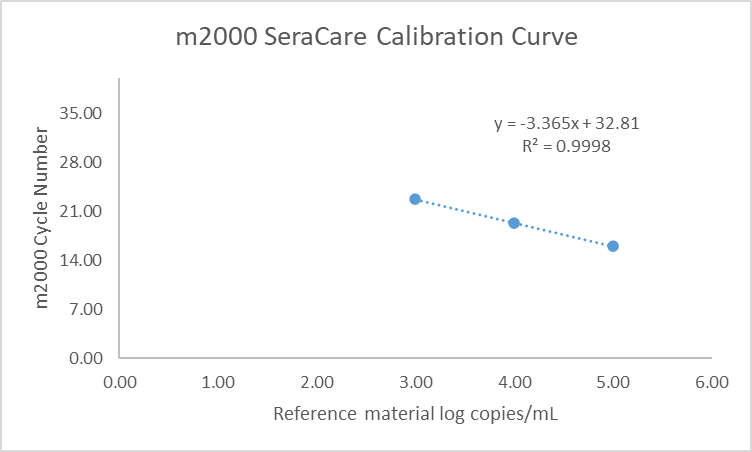


**Supplemental Methods Figure 1**. Calibration curve from commercial reference material spanning 3.0 to 5.0 log copies/mL concentrations.

**Supplemental Methods Table 2**. A pool of several positive patient samples from routine clinical testing was diluted and the SeraCare calibration curve was applied to the three samples with calculated concentrations overlapping the SeraCare material. After correcting for the dilution factor these three values were averaged to create an established value for the pooled positive sample and the six dilutions.

| fold-dilution | m2000 Cq | calculated log cp/ml | Established log cp/mL |
| --- | --- | --- | --- |
| 10 | 6.97 | 7.679 | 7.922 |
| 100 | 9.99 | 6.782 | 6.922 |
| 1000 | 13.17 | 5.837 | 5.922 |
| 10000 | 16.03 | ***4.987*** | 4.922 |
| 100000 | 19.83 | ***3.857*** | 3.922 |
| 1000000 | 23.01 | ***2.912*** | 2.922 |


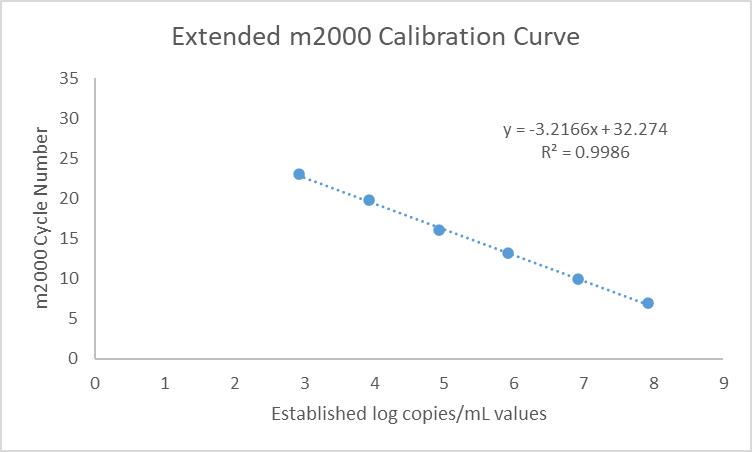


**Supplemental Methods Figure 2.** Extended Calibration curve based on established values of the dilution series of pooled positive patient samples.

**Supplemental Methods Table 3**. SeraCare reference material spiked into negative wastewater with extended calibration curve applied.

| Spike-in Concentration | replicate # | CN | log cp/mL |
| --- | --- | --- | --- |
| 100 cp/mL | 1 | 25.37 | 2.15 |
| 100 cp/mL | 2 | 25.85 | 2.00 |
| 100 cp/mL | 3 | 25.36 | 2.15 |
| 50 cp/mL | 1 | 27.77 | 1.40 |
| 50 cp/mL | 2 | 27.61 | 1.45 |
| 50 cp/mL | 3 | 27.51 | 1.48 |
| 25 cp/mL | 1 | 30.03 | 0.70 |
| 25 cp/mL | 2 | 26.78 | 1.71 |
| 25 cp/mL | 3 | 29.58 | 0.84 |


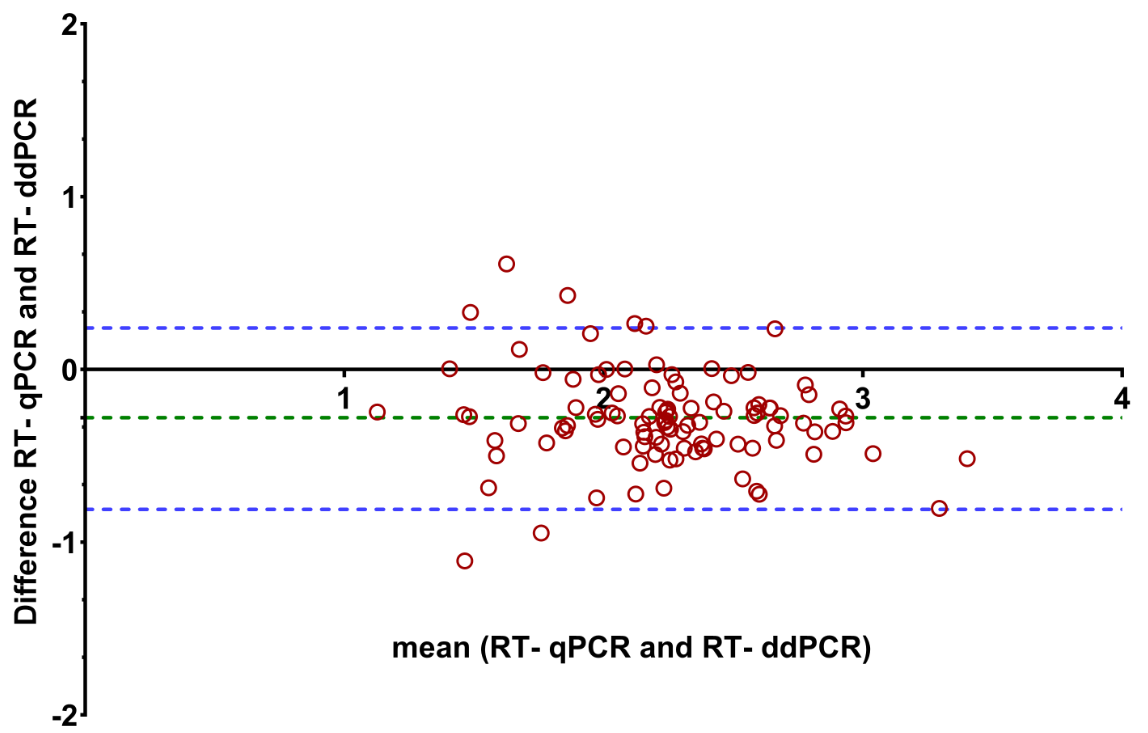


**Supplemental Figure 3:** **A Bland-Altman plot comparing the RT-qPCR and the RT-ddPCR assays.**The blue dotted lines represent the upper and lower limit of two standard deviations (± 2 SD) of the MEAN, and the green dotted line demonstrates the MEAN bias between the RT-qPCR and LDT RT-ddPCR.

**
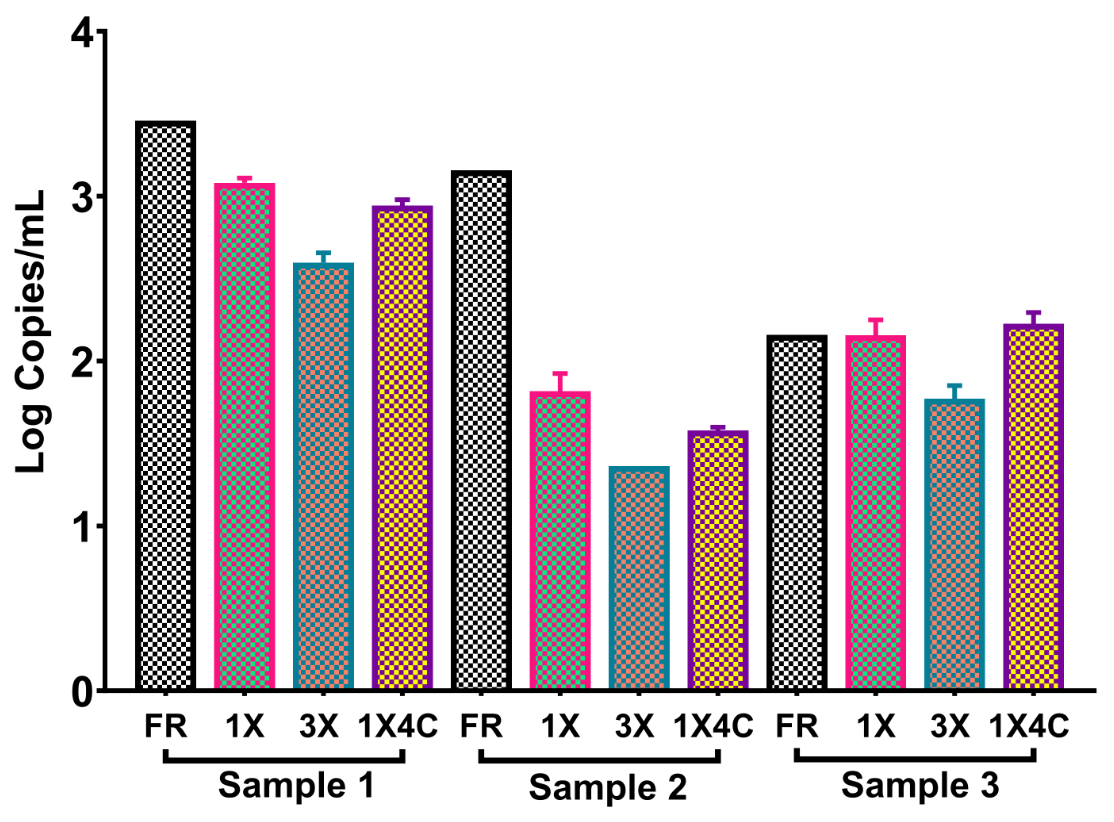
**

**Supplemental Figure 4:** **Raw data for wastewater sample stability studies. Three samples were tested in triplicate using the FDA EUA assay after different storage conditions.** FR = fresh, never frozen sample; 1X = 10 months of -80°C storage; 1X4C = 10 months of -80°C storage followed by refrigeration (4°C) for 24 hours; and 3X = 10 months of -80°C storage followed by three freeze-thaw cycles.
